# Impact of genetic similarity on imputation accuracy

**DOI:** 10.1186/s12863-015-0248-2

**Published:** 2015-07-22

**Authors:** Nab Raj Roshyara, Markus Scholz

**Affiliations:** Institute for Medical Informatics, Statistics and Epidemiology, University of Leipzig, Haertelstrasse 16-18, 04107 Leipzig, Germany; LIFE Center (Leipzig Interdisciplinary Research Cluster of Genetic Factors, Phenotypes and Environment), University of Leipzig, Philipp-Rosenthal Strasse 27, 04103 Leipzig, Germany

**Keywords:** Genotype imputation, Reference panel, Genetic similarity, F_ST, G_ST, SNP data, Imputation quality

## Abstract

**Background:**

Genotype imputation is a common technique in genetic research. Genetic similarity between target population and reference dataset is crucial for high-quality results. Although several reference panels are available, it is often not clear which is the most optimal for a particular target dataset to be imputed. Maximizing genetic similarity between study sample and intended reference panels may be the straight forward method for selecting the genetically best-matched reference. However, the impact of genetic similarity on imputation accuracy has not yet been studied in detail.

**Results:**

We performed a simulation study in 20 ethnic groups obtained from POPRES. High-quality SNPs were masked and re-imputed with MaCH, MaCH-minimac and IMPUTE2 using four different HapMap reference panels (CEU, CHB-JPT, MEX and YRI). Imputation accuracy was assessed by different statistics. Genetic similarity between ethnic groups and reference populations were measured by *F* -statistics (*F*_*ST*_) originally proposed by Wright and *G* -statistics (*G*_*ST*_) introduced by Nei and others. To assess the predictive power of these measures regarding imputation accuracy, we analysed relations between them and corresponding imputation accuracy scores. We found that population genetic distances between homogeneous reference and target populations were strongly linearly correlated with resulting imputation accuracies irrespective of considered distance measure, imputation accuracy measure, missingness and imputation software used. Possible exception was African population.

**Conclusion:**

Usage of *G*_*ST*_ or *F*_*ST*_-related measures for predicting the optimal reference panel for imputation frameworks relying on a specific reference is highly recommended. A cut-off of *G*_*ST*_ < 0.01 is recommended to achieve good imputation results for high-frequency variants and small data sets. The linear relationship is less pronounced for low-frequency variants for which we also observed a dependence of imputation accuracy on the number of polymorphic sites in the reference. We also show that the software specific measures MaCH-Rsq and IMPUTE-info must be interpreted with caution if the genetic distance of target and reference population is high.

**Electronic supplementary material:**

The online version of this article (doi:10.1186/s12863-015-0248-2) contains supplementary material, which is available to authorized users.

## Introduction

Genotype imputation is a common technique applied in the context of genome wide association (GWA) analysis. Typically, a set of densely genotyped samples is used as references to infer a large set of un-typed or missing markers in the target population. Although one has to deal with the uncertainty of genotypes derived by imputation, this procedure is nowadays standard since it makes large-scale genome-wide investigations feasible and cost effective. Furthermore, it enables meta-analysis by combining datasets genotyped at different platforms (e.g. Illumina versus Affymetrix arrays) [1]. It is also believed that genotype imputation improves the statistical power of genome wide association studies (GWAS) [2].

Moreover, imputation plays an essential role for the analysis of sequencing data [3]. Although, a dramatic cost reduction of next-generation sequencing technology was achieved, whole-genome sequencing of large study samples is still unaffordable. A way-out might be sequencing of a subset of individuals which could serve as an additional reference for imputation [4]. Strategies for selecting the individuals to be sequenced have been suggested recently [5]. These strategies consider genetic similarities between study population, subsets to be sequenced and the reference panel.

A number of different approaches have been suggested for building publically available reference panels that can maximize imputation accuracy. Some imputation software like IMPUTE2 [6] and MaCH-Admix [7] can exploit cosmopolitan references in order to optimize sequence similarity locally. However, other popular imputation frameworks (e.g. MaCH [8] and MaCH-minimac [9]) still rely on pre-selection of reference panels that are most closely matched with the ancestry of the study population For example, CEU is frequently used as imputation reference panel for European and European American samples, while CHB and JPT were chosen to impute samples from East Asian Populations [4].

Genetic distances like different *F*_*ST*_ measures [10–16] or principal component analysis [17] have been proposed to determine the genetic similarity between target and reference datasets. *F*-statistics were originally proposed by Wright to assess genetic structure of populations [10, 12]. Therefore, *F*_*ST*_ measures were constructed to evaluate the genetic distance between (homogeneous) populations, or in other words, the degree of genetic variance explained by ethnic sub-entities. Since first introduction of Wright’s *F*_*ST*_, a large variety of other *F*_*ST*_-related measures and corresponding estimators were proposed [11–18]. Nei [13, 14, 19] introduced the measure *G*_*ST*_ which is also frequently used for this purpose [15]. A few studies revealed that *F*_*ST*_-like measures calculated between target and reference populations correlate with imputation accuracy [20, 21].

However, it is still unclear how a reference panel with low genetic similarity affects the imputation accuracy. So far, no exact strategy (e.g. cut-offs for *F*_*ST*_-related measures) which could help us to select a well-suited reference panel has been proposed. To the best of our knowledge, there is no research on the relation between Nei’s *G*_*ST*_ and imputation accuracy. Therefore, in the present paper, we performed a simulation study to investigate the relationship of *G*_*ST*_ and other *F*_*ST*_-like measures and imputation accuracy obtained by three imputation frameworks: MaCH, MaCH-minimac and IMPUTE2. All these frameworks can be run with specific rather than cosmopolitan reference panels. Finally, we investigate the impact of missingness and frequency of variants on this relationship. All analyses were performed on the basis of the publically available dataset of POPRES [22].

## Materials and methods

### POPRES project

POPRES is a project fostering large Population Reference Samples of different ethnic origins [22]. The original POPRES project contains nearly 5,000 individuals of African-American, East Asian, South Asian, Mexican and European origin. Individuals included in the POPRES study are collected from different study groups all over the world. POPRES performed Genome-wide genotyping of these individuals on the Affymetrix (Mountain View, CA) GeneChip 500 K Array set with the published protocol for 96-well-plate format. Sample collection and methods for POPRES are described elsewhere [22]. The datasets used for the present analyses were obtained from dbGaP [23] through dbGaP accession number phs000145.v4.p2.

### Datasets

We considered chromosome 22 from the POPRES dataset for our research. This dataset originally consisted of 5,637 SNPs measured in individuals from 35 different populations. To avoid biases due to different sample sizes and to include as many populations as possible into our analysis, we considered an equal number of individuals for each sub-population (N = 40). If more than 40 individuals are available, a random sample of N = 40 was drawn to rule out effects caused by differing sample sizes. Population groups with less than 40 members were discarded resulting in a total of 20 different ethnic subsets, namely 15 populations of Caucasian origin: Australian, Canadians, German, French, Swiss-French, Swiss-German, Swiss, Italian, Spanish, Irish, British, Belgish, Portuguese, former Yugoslavia, a mixed group of east European origin (a mixture of people from Czech-republic, Hungary, Poland); two populations of South-Asian origin: Indians and Punjabis, one east-Asian population: Japanese, one Mexican population: Mexican, and finally, a mixed-population of African-Americans (AfAm). Study populations which do not match very closely with the available HapMap references CEU, JPT, CHB, YRI (see below) were supposed to indicate the impact of imperfect reference panels on the target populations. This might be applicable for the following populations: Indian, Punjabis, Yugoslavians, East-EU, Portuguese and African-Americans. Target populations like Europeans are abbreviated by EU, East European populations (eastEU and Yugoslavia) by EEU, South Asians (Punjabi and India) as SASI, Japanese by Jap, Mexican by MEX, South European (Italian, Portuguese) by SEU and African Americans by AfAm.

### Reference Panel

1000 Genomes datasets were based on low depth whole genome sequencing data and are generally considered to have lower accuracy than HapMap data. Thus we considered HapMap3 [24] reference panels (NCBI Build 36) to impute the above mentioned populations. Four different pre-formatted reference panels: CEU, YRI, MEX and JPT + CHB provided by the MaCH-developers [25] and IMPUTE-developers [26] were considered. In a full-factorial design, we imputed our target populations with these reference panels.

### Strand verification of SNPs

Genomic assembly of the original POPRES data was identical to Affymetrix release 25 NSP25 and STY25 and the corresponding rs-IDs were identified by NCBI build “b36” with UCSC version “hg18”. Strand alignment between study sample and reference data was performed using fcGENE [27] and PLINK [28]. SNPs with ambiguous strands and SNPs which could not be found in the HapMap3 reference panel were removed. In total 1,014 SNPs could not be matched to HapMap3 reference panels and were excluded. 4,623 SNPs overlapping with HapMap reference panel remained for further analyses.

### Selection of good-quality (GQ) SNPs

Good quality (GQ)-SNPs were selected with stringent filtering criteria of genotype quality. These GQ SNPs were then assumed to express true genotypes for our experimental study. In analogy to our previous research [29], we masked these SNPs and re-imputed them to evaluate the imputation accuracy as explained in the next section. More precisely, we compared the posterior genotype probability distributions produced by the imputation software with the corresponding true genotypes. To select GQ SNPs, we apply the following quality criteria: average call rate (CR averaged over all populations > =95 %), average minor allele frequency (MAF averaged over all populations > =0.1) and p-values of stratified Hardy Weinberg Equilibrium Test (p (HWE) > =10e-2). Since the samples were from multiple ethnic group, we used exact stratified test of HWE [30]. A total of 457 SNPs passed these quality criteria.

### Masking Process

We performed the masking process in two phases. First, we masked all good quality SNPs and imputed them with the imputation frameworks: MaCH, MaCH-minimac and IMPUTE2. We also considered additional scenarios where we masked only 70 % and 50 % of the previously selected good quality SNPs. This type of masking was performed in such a way that all SNPs masked in the lower percentage of missingness were also masked in the higher percentages of missingness. The first type of masking (100 %) was used to investigate the relationships between *G*_*ST*_ and *F*_*ST*_-related scores and corresponding imputation accuracy. The second type of masking was used to study the impact of different degrees of missingness on these relations. For this purpose, we only compared the 50 % GQ SNPs which were masked in all three missingness scenarios to avoid bias introduced by SNP selections.

### Imputation

Imputations were performed separately for each of the previously mentioned sets of populations combined with any of the four reference panels. As suggested by MaCH developers, imputation with this software was performed in two steps: In the first step, imputation error rate and recombination rate were estimated. These two model parameters were determined by running the “greedy” algorithm for 100 iterations and were used in the second step to determine the transition probabilities of the underlying Hidden Markov Model [8]. In the second step, the most likely genotype probability distributions of each genotype at each individual and the imputation quality measured by the software specific Rsq score were determined. Commands used for MaCH-imputation are provided in Additional file 1. The relative performance of imputation methods differ greatly as a function of sample sizes, marker densities and parameters of the algorithm such as the number of EM iterations. Therefore, the same standard parameter settings were used for each imputation process.

Imputation with MaCH-minimac was also performed in two steps. In the first step, MaCH was used to predict the haplotypes of the study data sets, and then, minimac was used to calculate the posterior probabilities of the genotypes using these haplotypes.

As suggested by the software developer, imputation with IMPUTE2 was performed in a segmented way by defining different genomic intervals approximately of size 5 MB. An internal buffer region of 250 kb on both sides of the analysis interval was used to avoid the margin effects of chromosome segmentation.

After imputation, we compared the estimated posterior distribution with the measured genotypes as explained below. Considering four reference panels, 20 target populations, two missing scenarios and three software packages, a total of 480 imputations were performed.

### Assessment of imputation accuracy

A common strategy for determining imputation performance is to compare true genotypes (genotypes measured by a technique with high confidence or consensus genotypes) with corresponding imputation results. Here, we directly compared the posterior distributions of our re-imputed GQ-SNPs with corresponding measured genotypes applying our recently proposed Hellinger and SEN score [31]. Both measures are platform independent. While Hellinger score measures the distance of imputed and measured genotype probability distribution, SEN score is maximal if their expectations are identical. Thus, SEN essentially compares gene doses. Cut-offs of 0.95 for SEN score and 0.45 for Hellinger score respectively were considered as indicators of good imputation accuracy (for motivation, see also Fig. 2 below). We also analysed imputation accuracy using the software specific measures MaCH-Rsq and IMPUTE-info determined during the imputation process. MaCH-Rsq measure is basically defined as the ratio of the empirically observed variance of the allele dosage to the expected binomial variance under Hardy-Weinberg equilibrium [32]. Similarly, IMPUTE-info score is the relative statistical information about the SNP allele frequency derived from the imputed data [33]. These two software-specific measures are defined at SNP-level and are useful to assess imputation quality of SNPs for which no measurements are available. These scores are widely applied to remove SNPs with low imputation accuracy during post-imputation quality control.

While Hellinger and SEN scores assess agreement of imputed and observed genotypes individually, the software specific measures assess imputation quality for entire SNPs, i.e. cannot be interpreted for single genotypes.

### Estimation of *G*_*ST*_ and other *F*_*ST*_-related measures

Nei’s *G*_*ST*_ is defined as the ratio of average gene diversity within subpopulations and the gene diversity of the total pooled population:

$${G}_{ST}=\frac{{D^{\hbox{'}}}_{ST}}{H_T},$$

where *H*_*T*_ is the heterozygosity expected under Hardy-Weinberg equilibrium for the total pooled population and *D*’_*ST*_ is the average gene diversity between the subpopulations [13, 14, 19]. For two-allelic markers, Bhatia et al. [16] recommended the estimator of *G*_*ST*_ at any particular *k*^*th*^ marker as:

$$\begin{array}{l}{G}_{ST}^{\left[k\right]}=\frac{D^k}{H_T^k},{D}^k={\left({p}_1^k-{p}_2^k\right)}^2, \\ {}\kern2.64em {H}_T^k=2{p}_{avg}^k\left(1-{p}_{avg}^k\right),\\ {}\kern3.48em {p}_{avg}^k=\frac{p_1^k+{p}_2^k}{2}\end{array}$$

where *p*_1_^*k*^ and *p*_2_^*k*^ are the allele frequencies of the reference allele at the *k*^*th*^ marker in the two populations. To calculate *G*_*ST*_ between two population groups genotyped at N markers, one can use the formula:

$${G}_{ST}=\frac{{\displaystyle {\sum}_{k=1}^N}{D}^k}{{\displaystyle {\sum}_{k=1}^N}{H}_T^k}$$

Computation of pair-wise *G*_*ST*_ between any two population is implemented in the most recent version of fcGENE [27]. Small values of *G*_*ST*_ indicate that allele frequencies between the two populations are similar, i.e. the genetic distance between them is small.

Regarding *F*_*ST*_-related measures, we considered *F*_*ST*_^*R*^ described in the work of Reich *et al.* [17], and implemented in the program EIGENSOFT, in which a block-jack knife procedure is used to estimate the standard error of *F*_*ST*_^*R*^. For any *k*^*th*^ Marker, *F*_*ST*_^*R*^ is calculated as

$$\begin{array}{l}\kern1.68em {F}_{ST}^{R\left[k\right]}=\frac{N^k}{D^k}\\ {}{N}^k={\left(\frac{a_1}{m_1}-\frac{a_2}{m_2}\right)}^2-\frac{h_1}{m_1}-\frac{h_2}{m_2}\\ {}\kern0.84em {D}^k={N}^k+{h}_1+{h}_2,\end{array}$$

where a_1_ and a_2_ are the specific allele counts and m_1_ and m_2_ are the total allele counts of the marker in two population. Heterozygosities of the markers are 2 h_1_ and 2 h_2_, with ${\mathrm{h}}_1=\frac{{\mathrm{a}}_1\left({\mathrm{n}}_1-{\mathrm{a}}_1\right)}{{\mathrm{n}}_1\left({\mathrm{n}}_1-1\right)}$, ${\mathrm{h}}_2=\frac{{\mathrm{a}}_2\left({\mathrm{n}}_2-2\right)}{{\mathrm{n}}_2\left({\mathrm{n}}_2-1\right)}$ respectively. Let n_1_ and n_2_ be the numbers of individuals genotyped in the two populations at the *k*^*th*^ marker. The allele counts a_1_ and a_2_ and the total allele counts m_1_ and m_2_ can be determined as a_1_ = 2u_1_ + v_1_, a_2_ = 2u_2_ + v_2_, m_1_ = 2n_1_, m_2_ = 2n_2_, where u_1_ and v_1_, and u_2_ and v_2_ are the counts of homozygotes and heterozygotes in the first, and in the second population respectively. Now if there are N markers genotyped in each population, an unbiased estimator of F_ST_ can be defined as

$${\mathrm{F}}_{\mathrm{ST}}^{\mathrm{R}}=\frac{{\displaystyle {\sum}_{\mathrm{k}=1}^{\mathrm{N}}}{\mathrm{N}}^{\mathrm{k}}}{{\displaystyle {\sum}_{\mathrm{k}=1}^{\mathrm{N}}}{\mathrm{D}}^{\mathrm{k}}}$$

In order to compare the relative performance of different *F*_*ST*_-related measures in predicting imputation accuracy, we also computed the original and modified estimators of *F*_*ST*_ (denoted by *F*_*ST*_^*WC*^ and *F*_*ST*_^*mWC*^) proposed by Weir and Cockerham [11]. *F*_*ST*_^*WC*^ between two population was calculated as follows

$${F}_{ST}^{WC}=\frac{{\displaystyle {\sum}_{k=1}^N}{N}^k}{{\displaystyle {\sum}_{k=1}^N}{D}^k}$$

where

$$\begin{array}{c}\hfill {N}^k={s}^2-\frac{1}{2\overline{n}-1}\left[\overline{p}\ \left(1-\overline{p}\right) - \frac{\ {s}^2}{2}-\frac{\overline{h}}{4}\right]\hfill \\ {}\hfill {D}^k=\overline{p}\ \left(1-\overline{p}\right) + \frac{\ {s}^2}{2}\hfill \\ {}\hfill \begin{array}{l}{s}^2=\frac{n_1\left({p}_1^k-\overline{p}\right)+{n}_2\left({p}_2^k-\overline{p}\right)}{\overline{n}},\overline{p}=\frac{\left({p}_1^k+{p}_2^k\right)}{2}\\ {}\\ {}\overline{h}=\frac{2{n}_1{p}_1^k\left(1-{p}_1^k\right)+2{n}_2{p}_2^k\left(1-{p}_2^k\right)}{2\overline{n}},\overline{n}=\frac{\left({n}_1+{n}_2\right)}{2}\end{array}\hfill \end{array}$$

A modified estimator *F*_*ST*_^*mWC*^ of Weir and Cockerham’s *F*_*ST*_ is defined as follows [16]:

$$\begin{array}{c}\hfill {F}_{ST}^{mWC}=\frac{{\displaystyle {\sum}_{k=1}^N}{N}^k}{{\displaystyle {\sum}_{k=1}^N}{D}^k}\hfill \\ {}\hfill {N}^k={\left({p}_1^k-{p}_2^k\right)}^2\hfill \\ {}\hfill {D}^k=\left({p}_1^k-{p}_2^k\right)+\frac{2}{n_1+{n}_2}\left[{n}_1{p}_1^k\left(1-{p}_1^k\right)+{n}_2{p}_2^k\left(1-{p}_2^k\right)\right]\hfill \end{array}$$

These formula of *F*_*ST*_^*WC*^ and *F*_*ST*_^*mWC*^ are also implemented in fcGENE. While computing Weir and Cockerham *F*_*ST*_ and Nei’s *G*_*ST*_, we used the same reference alleles throughout all populations.

In previous studies [20], *F*_*ST*_ was computed for individual SNPs and then averaged across SNPs. However these *F*_*ST*_ estimators does not account for haplotype diversity very well [34]. Therefore, in our formula all quantities were averaged over all SNPs first, and then, *F*_*ST*_ is calculated. This estimate is more precise, i.e. results in smaller standard errors as pointed out in [17, 35].

### Correlation statistics

Calculation of imputation quality scores is based on GQ SNPs masked prior to imputation. After calculation of *G*_*ST*_ and other *F*_*ST*_ related measures between POPRES populations and the four reference panels considered, we compared population distances with corresponding imputation accuracy scores. Different scatter plots were generated using the R-package ‘ggplot2’ [36] allowing to construct smoothed curves of non-linear relationships. To determine the correlation among *G*_*ST*_ and other *F*_*ST*_-related measures, we used Kendall’s rank correlation coefficient (Kendall’s tau coefficient) [37], which measures the similarity of the ordering of the data to be compared.

## Results

### Comparison of measures of genetic distance between populations

**Fig. 1:**
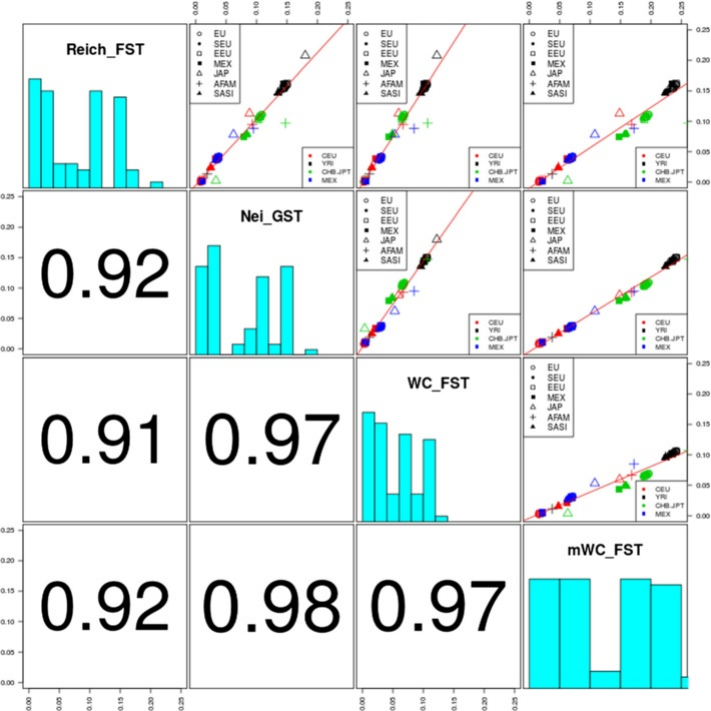
Scatter plot and correlations of different measures of population distance determined on the basis of pair-wise comparisons of POPRES populations and HapMap reference panels. In the lower triangle, Kendall’s rank correlations are presented. In the upper triangle, symbols represent POPRES population while colors represent the reference panel to which the distance is calculated. A regression line is added to each scatter plot. At the diagonal, histograms of measures are added

First, we compared our different measures of population distances derived from all pair-wise comparisons of POPRES data subsets and reference populations. Results can be found in Fig. 1. The figure shows that Nei’s *G*_*ST*_ is almost equivalent to the other *F*_*ST*_-related measures, i.e. a strong linear relationship is observed. *F*_*ST*_^*WC*^ and *F*_*ST*_^*mWC*^ are better correlated with *G*_*ST*_ than *F*_*ST*_^*R*^. In the scatter plot of *G*_*ST*_ and *F*_*ST*_^*R*^, the strongest deviation from the linear trend is caused by the population group AfAm with reference panel CHB. JPT. We investigated the cause of this deviation and found that low-frequencyvariants (SNPs with MAF ≤ 0.05) strongly influence *F*_*ST*_^*R*^ while *G*_*ST*_ is robust (see Additional file 1: Table S1).

### Characterization of measures of imputation accuracy

**Fig. 2:**
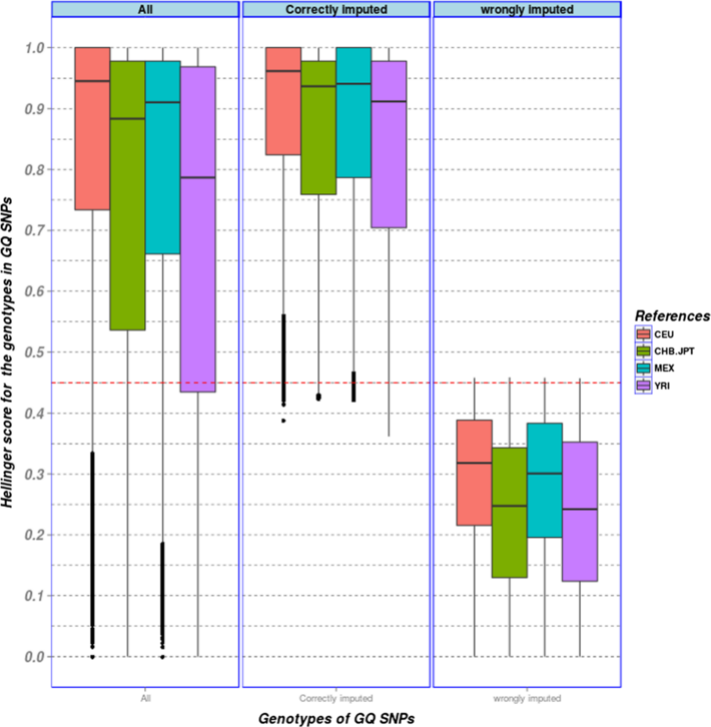
Box plot of Hellinger scores of Germanic target population obtained from MaCH- imputation with four different reference panels. Results for correctly and wrongly imputed SNPs based on best-guess genotypes are presented separately. CEU achieves highest Hellinger scores for all, correctly and incorrectly imputed genotypes, i.e. performed best among reference panels. As one can see, applying a threshold of 0.45 for Hellinger scores almost ensures that the best-guess genotype is correct

Next, we analysed the accuracy scores obtained from imputing each of the 20 target populations with any of the four reference panels using the three imputation frameworks. As an example, distribution of imputation accuracy scores of target population from Germany imputed with the four different reference panels applying MaCH are displayed in Fig. 2.

As expected, the target population from Germany is best imputed with the genetically closest reference panel “CEU” followed by “MEX”, “CHB. JPT” and “YRI”. A cut-off for Hellinger score of 0.45 almost perfectly separates correctly and incorrectly imputed genotypes. We obtained similar trends for other target populations as for example “AfAm” was best imputed with the ethnically closest reference panel “YRI” (see Additional file 1: Figure S1) although overall imputation yield is substantially reduced in this population.

**Fig. 3:**
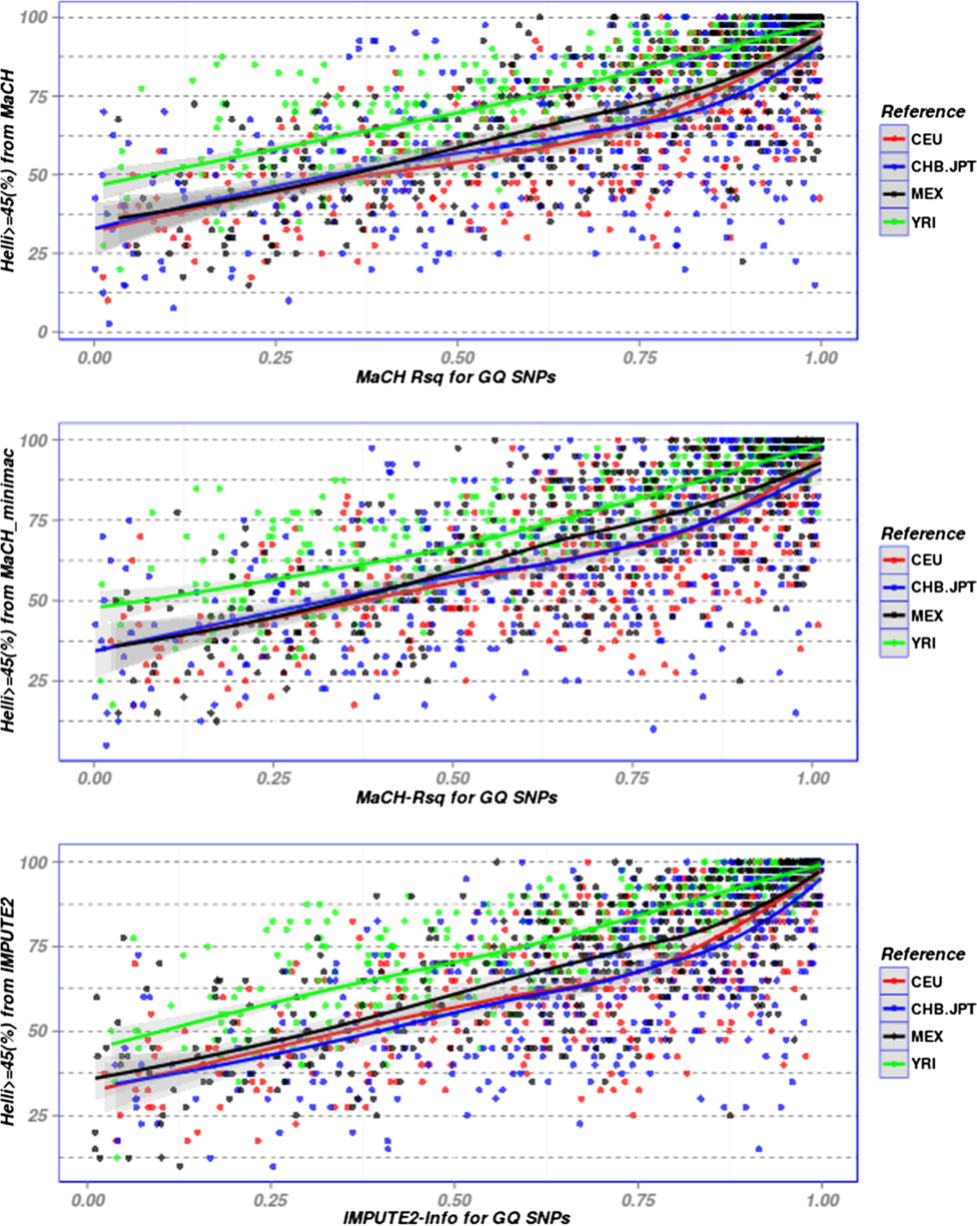
Scatter plot between Rsq-score/Info-score and average Hellinger score of GQ SNPs. In the three sub-figures, imputation results of AfAm obtained with the three software packages MaCH, MaCH-minimac and IMPUTE2 are presented. Color represents results after imputation with one of the four reference panels CEU, CHB_JPT, MEX and YRI. Results are smoothed by loess estimators. For a particular value of MaCH-Rsq/IMPUTE-info, Hellinger scores obtained by using a genetically similar reference panel are higher than those obtained from mismatching reference panels (p-value < 0.001 for all three scenarios, based on a regression analysis considering the software-specific score as covariable)

MaCH-Rsq and IMPUTE-info scores are typically used for post-imputation quality control. These scores are essentially based on the ratio of sample variance of allele frequency during imputation and its expected variance under Hardy-Weinberg equilibrium. The expected variance depends on the allelic frequency of the corresponding SNP in the reference panel considered. Thus, in contrast to Hellinger or SEN score, imputation accuracy determined by MaCH-Rsq and IMPUTE-info scores depends on the reference sample used. We studied the relationship between MaCH-Rsq/ IMPUTE-info score and the average Hellinger score of GQ SNPs. Exemplarily, results of AfAm imputed with the four different reference panels are shown in Fig. 3. We observed a monotonous relationship between average Hellinger score and Mach-Rsq/IMPUTE-info irrespective of the target dataset or reference used. However, it turned out that for given Mach-Rsq/IMPUTE-info values, corresponding average Hellinger scores were higher for genetically matching reference panels compared to mismatching reference panels. This behaviour is especially pronounced for AfAm population where reference panels other than YRI result in particularly low average Hellinger scores even if corresponding MaCH-Rsq/ IMPUTE-info values are high (Fig. 3). This indicates that MaCH-Rsq/IMPUTE-info values measure imputation quality accurately only if a genetically matching reference is used.

### Correlation of Nei’s *G*_*ST*_ and *F*_*ST*_-related scores with imputation accuracy

**Fig. 4:**
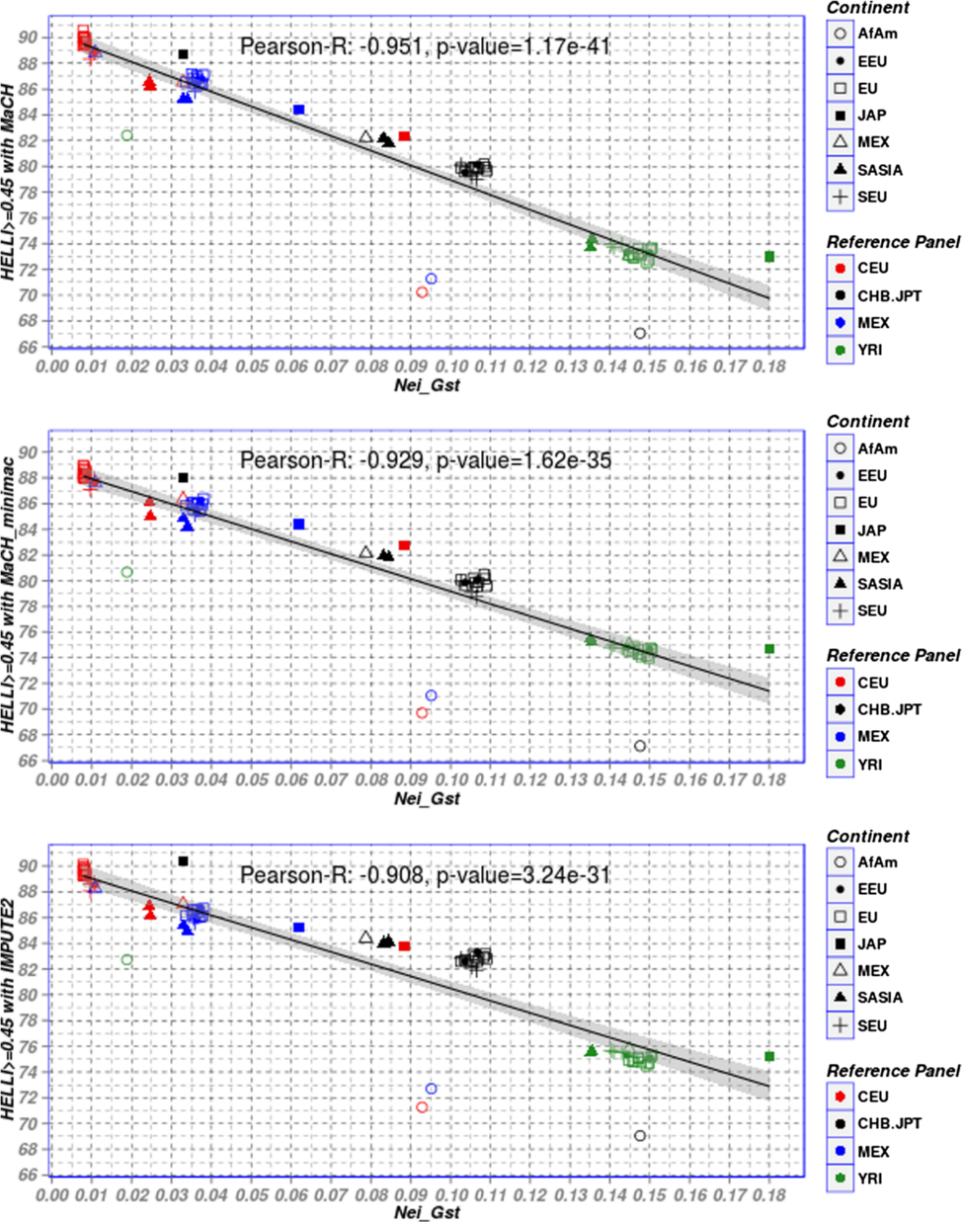
Scatter plot of Nei’s G_ST_ and percentages of well imputed GQ genotypes (Hellinger score ≥ 0.45) for all combinations of target and reference populations imputed with the three software packages considered. Color decodes reference panel while symbol represents the POPRES population considered. Pearson’s correlation coefficients and the p-values obtained from computing a test of the correlation being zero are also described

We investigated the relationship between *G*_*ST*_ and *F*_*ST*_-related scores and imputation accuracy. In view of the good correlation of *G*_*ST*_ and *F*_*ST*_-related scores, we focus on *G*_*ST*_ in the following. Since good Hellinger scores (≥0.45) represent correctly imputed genotypes in most cases, percentages of genotypes with Hellinger score ≥0.45 in dependence on *G*_*ST*_ serve as primary outcome of our analyses. Results can be found in Fig. 4 showing the scatter plot between pair-wise Nei’s *G*_*ST*_ and the percentage of genotypes with Hellinger score ≥0.45 for all target populations imputed with the four reference panels.

We observed an almost linear relationship between *G*_*ST*_ and this measure of imputation quality for all three software packages. Pearson’s correlation coefficients between *G*_*ST*_ and imputation quality are -0.95, -0.93 and -0.91 for MaCH, Mach-minimac and IMPUTE2, respectively. We conclude that *G*_*ST*_ is a good predictor of imputation accuracy for all type of imputation frameworks used under the best-matching policy for selecting a reference panel. Small values of *G*_*ST*_ imply high imputation accuracies and vice versa. Only AfAm is an outlier of this relationship resulting in particularly low imputation quality even if YRI as best matching reference panel was used.

This outlying behaviour of AfAm was consistently observed for all three software packages considered. To analyse whether the sample size has an impact, we additionally considered the complete AfAm sample of POPRES with N = 252. For the reference sample YRI the results of all software packages were slightly improved, but we also observed a small reduction in *G*_*ST*_. For the other reference panels, we observed no difference to the results of MaCH-minimac and IMPUTE2 obtained for the original sample (N = 40). However, for MaCH we observed a small deterioration of Hellinger score for the larger sample. Results are shown in Additional file 1: Figures S10 and S11. We conclude that sample size alone does not explain the observed outlying behaviour of AfAm.

**Fig. 5:**
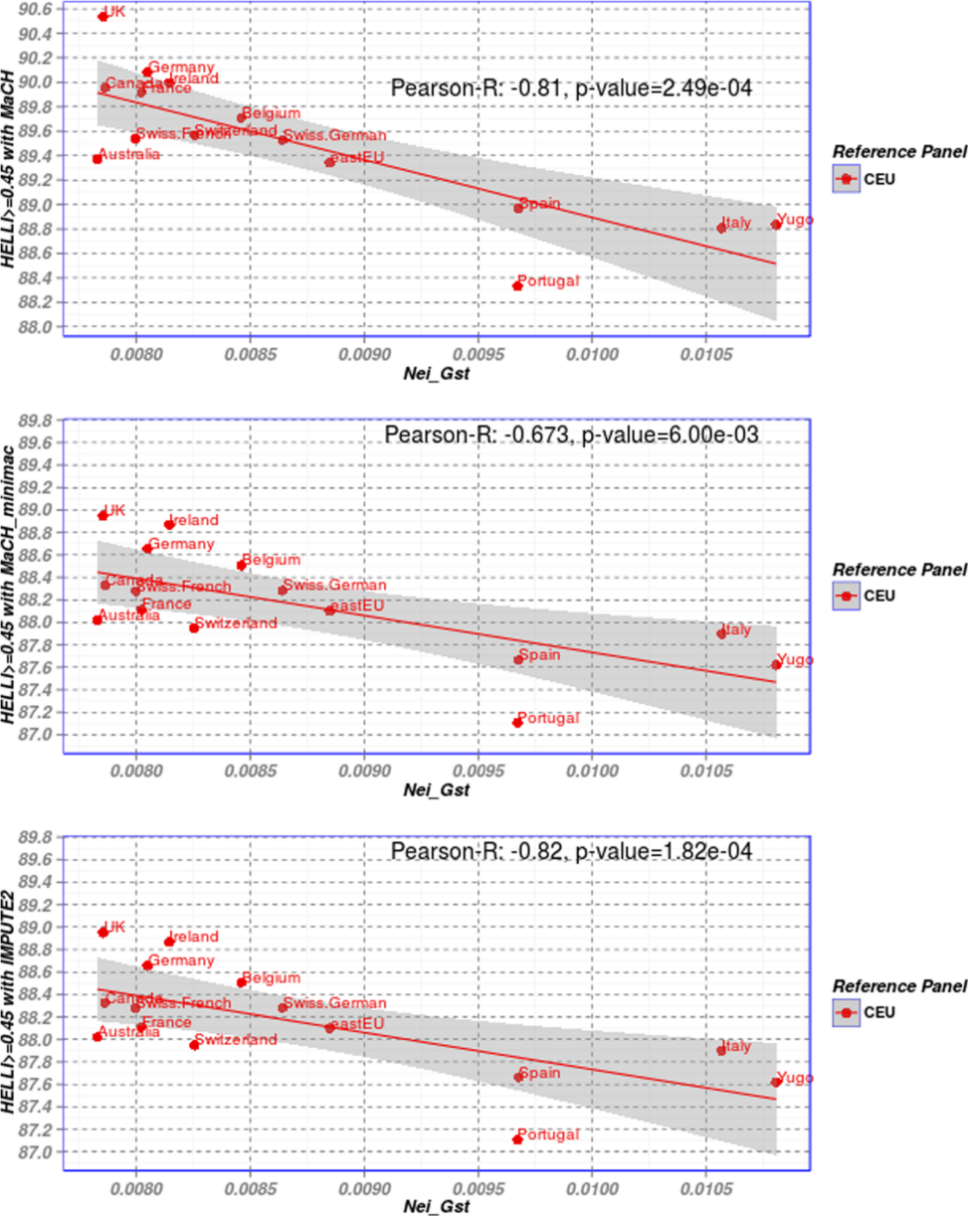
Scatter plot of Nei’s G_ST_ and percentage of genotypes with Hellinger score ≥0.45 for European populations imputed with CEU reference panel. Still, a linear relationship can be observed. Pearson’s correlation coefficients and the p-values obtained from computing a test of the correlation being zero are also described

Correlation between MaCH-Rsq/IMPUTE-info score and *G*_*ST*_ showed similar behaviour (Additional file 1: Figure S2). Moreover, *G*_*ST*_ was also highly correlated with the percentage of genotypes with SEN ≥0.95 as shown in Additional file 1: Figure S3. Analyzing the relationship between *G*_*ST*_and imputation accuracy in more detail, it can be recommend that *G*_*ST*_ between the target and reference population should be smaller than 0.04 to achieve a yield of at least 87 % well-imputed common SNPs. AfAm is an exception of this rule. In our data, good imputation results with about 90 % correctly imputed GQ SNPs are obtained if the value of *G*_*ST*_ is less than 0.01. Since the largest set of POPRES populations are from Europe, we performed a more detailed analysis of this sub-group (Fig. 5). Interestingly, using CEU as reference, we obtained again a trend towards lower imputation accuracy for larger values of *G*_*ST*_. Notably, the populations from east and south Europe show somewhat lower yield of well imputed genotypes than those from Central and Western Europe.

Scatter plots of other measures of population distance (e.g. *F*_*ST*_^*R*^) and imputation accuracy are similar. Results for F_ST_^R^  can be found in Additional file 1: Figures S4 and S5.

We also computed correlation coefficients between *G*_*ST*_ and imputation accuracy of the 20 POPRES samples in dependence on reference, software and measure of imputation quality (Table 1). A strong linear trend was observed for all of these scenarios.

**Table 1:** Pearson correlation between Nei’s *G*_*ST*_ and imputation quality scores of the 20 POPRES populations in dependence on reference panel, imputation software and measure of imputation quality, CIG = correctly imputed genotypes

Reference	Software	HELLI > =0.45 (%)	SEN > =0.45 (%)	CIG (%)	Rsq/Info mean	Rsq/Info > =0.8 (%)
CEU	MaCH	−0.907	−0.911	−0.902	−0.959	−0.930
	MaCH_minimac	−0.870	−0.871	−0.868	−0.914	−0.865
	IMPUTE2	−0.877	−0.888	−0.876	−0.943	−0.898
YRI	MaCH	−0.962	−0.969	−0.966	−0.975	−0.849
	MaCH_minimac	−0.950	−0.961	−0.953	−0.971	−0.620
	IMPUTE2	−0.953	−0.966	−0.956	−0.969	−0.507
CHB. JPT	MaCH	−0.907	−0.905	−0.910	−0.767	−0.739
	MaCH_minimac	−0.892	−0.893	−0.896	−0.767	−0.785
	IMPUTE2	−0.853	−0.852	−0.856	−0.784	−0.806
MEX	MaCH	−0.917	−0.915	−0.914	−0.946	−0.923
	MaCH_minimac	−0.898	−0.892	−0.898	−0.908	−0.882
	IMPUTE2	−0.898	−0.902	−0.901	−0.932	−0.904

By estimating the association between *G*_*ST*_ and imputation accuracy score and by computing a test of the correlation being zero, we got p-value < 9.52e-13 for all three scenarios

### Dependence on degree of missingness

**Fig. 6:**
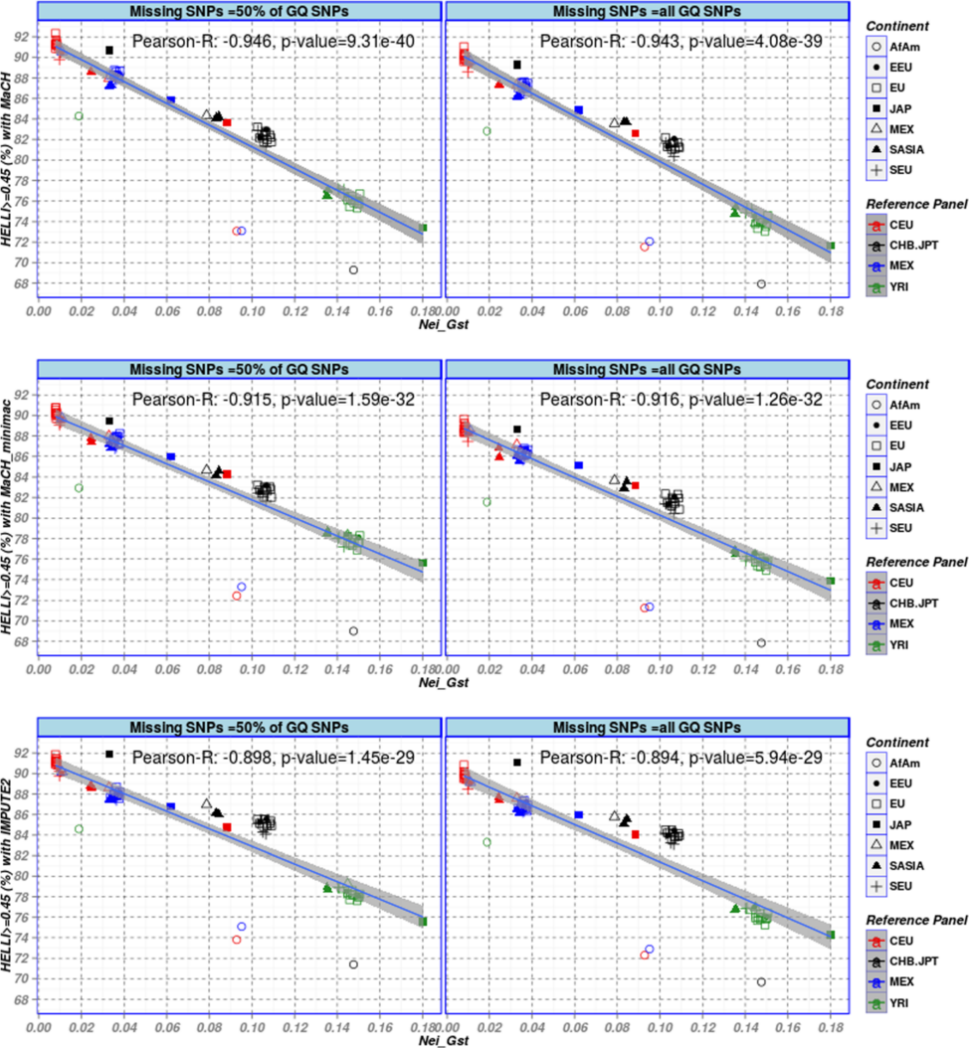
Scatter plot of *G*_*ST*_ and percentages of well imputed genotypes (Hellinger score ≥ 0.45) at different degrees of missing. Performance of the 50 % GQ SNPs missing in all scenarios was analysed. While the degree of missingness has a clear impact on imputation accuracy, the linear trend between *G*_*ST*_ and imputation accuracy is essentially preserved. Pearson’s correlation coefficients and the p-values obtained from computing a test of the correlation being zero are also described

In order to study the impact of different degrees of missingness on the relation between imputation accuracy and *F*_*ST*_-related measures, we compared *G*_*ST*_, *F*_*ST*_^*R*^, *F*_*ST*_^*WC*^ and *F*_*ST*_^*mWC*^ with imputation accuracies at different degrees of missingness. Although, degree of missingness has a clear impact on overall imputation accuracy, it turned out that this has only a marginal impact on the observed linear relationship between population distance and imputation accuracy. Fig. 6 shows the results for Nei’s *G*_*ST*_. Results of other accuracy endpoints and measures of genetic distance are similar and can be found in the supplement material (Additional file 1: Figures S6, S7 and S8).

### Impact on low frequency variants

**Fig. 7:**
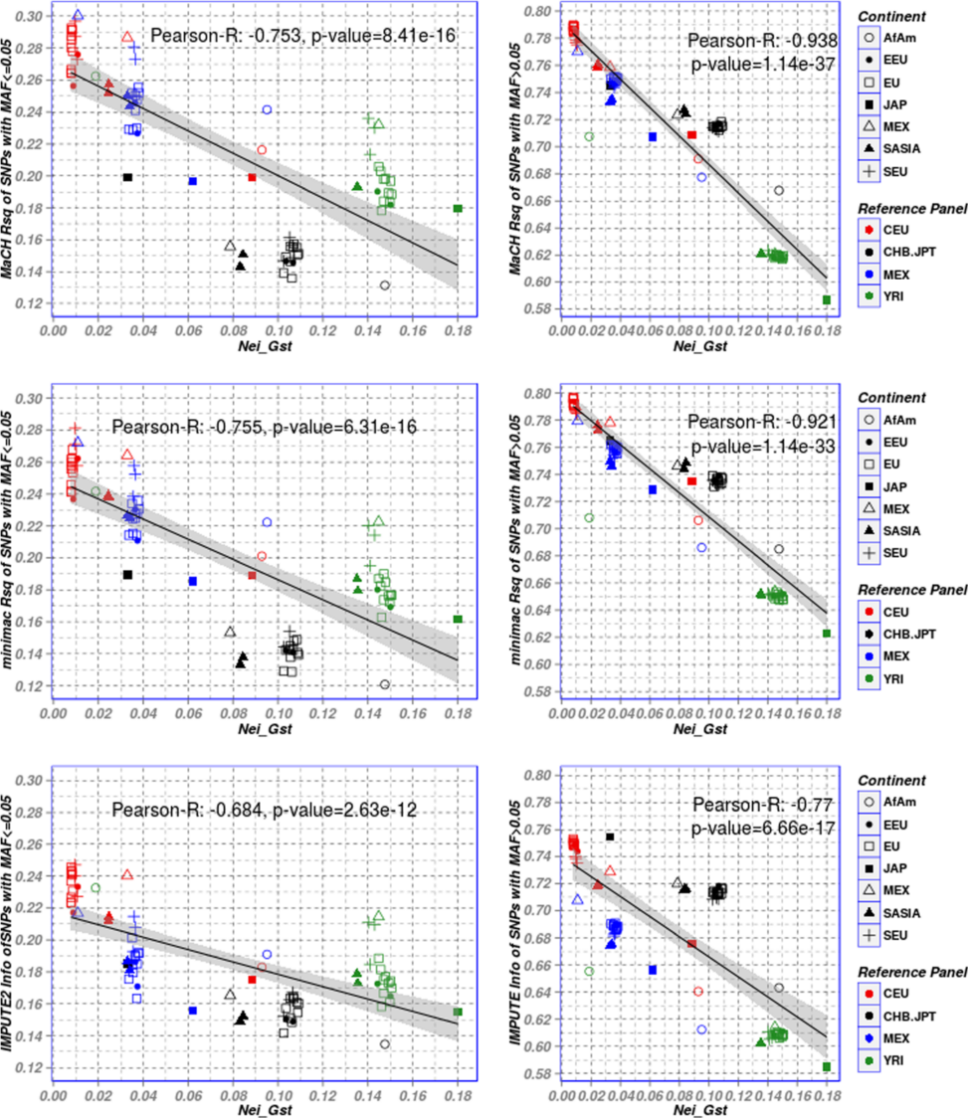
Scatter plot of *G*_*ST*_ and average Rsq-score/Info-scores of low-frequency variants (left panels) versus common variants (right panels). We present the results of the three imputation frameworks MaCH, MaCH-minimac and IMPUTE2. For low-frequency variants, both, overall yield of well-imputed SNPs and correlation between *G*_*ST*_ and imputation accuracy are reduced. Pearson’s correlation coefficients and the p-values obtained from computing a test of the correlation being zero are also described

Finally, we analyzed how *G*_*ST*_ and other *F*_*ST*_-related scores correlate with imputation accuracies of low-frequency variants (MAF ≤ 5 %). Fig. 7 shows the results for *G*_*ST*_ and the software-specific measures of imputation accuracy. As expected, the overall yield of well-imputed low-frequency variants is lower than for the common variants. Moreover, the correlation of *G*_*ST*_ and imputation accuracy is also markedly reduced compared to the common variants. Correlation between *F*_*ST*_ and the software-specific measures of low-frequency and common variants is displayed in Additional file 1: Figure S9 showing similar results.

## Discussion

Imputation of un-typed SNPs or missing genotypes is a common technique in genome-wide analyses. However, accuracy of imputation is difficult to predict as it depends on a variety of factors including pre-imputation quality control, genetic similarity of reference and target population, and its haplotype structure. We recently performed a comprehensive simulation study analyzing the effect of pre-imputation quality control on accuracy of imputation [29]. In the present paper, we studied the impact of reference panels on imputation accuracy. For this purpose, we considered the three software packages MaCH, MaCH-minimac and IMPUTE2 which can be run with a population-specific reference panel. Other approaches relying on mixed reference panels were proposed recently [21, 38] circumventing the issue of selecting appropriate references (e.g. IMPUTE2 [39], MaCH-Admix [7]). However, previous researches [29, 46] and our own results (submitted) showed that such algorithms could reduce imputation quality compared to frameworks relying on specific references. Thus, software packages like MaCH or MaCH-minimac are still frequently in use [4, 40–44]. It is beyond any doubt that in this case, the reference panel should ethnically match with the target population as best as possible so that it can represent the haplotype structures of the individuals in the target population. Consequently, for these imputation frameworks, it is recommended to choose a reference panel best-matched with the ancestry of the target population. This can be achieved for example by analysing measures of genetic distances between target and reference populations [20, 45]. However the relation between genetic distance and imputation accuracy is not completely understood and requires further research.

In order to analyse this issue in more detail, we performed a simulation study on the basis of ethnic sub-samples of the publically available POPRES panel [22]. A total of 20 target datasets were considered. However, samples were small regarding both, number of SNPs and individuals. This implies that our results may be valid only for small or medium-sized data sets.

Four ethnic reference data sets derived from HapMap3 (NCBI Build 36) were considered, namely CEU, YRI, MEX and JPT + CHB. Reference data are provided through the home pages of MaCH- and IMPUTE-software developers. These four reference data sets allowed us investigating the dependence of imputation accuracy on genetic similarity between target and reference panels for a higher number of combinations. In our paper, we focused on imputation of high-frequency variants. Although relying on HapMap3 reference might be a limitation of our study, we expect that the results for these variants are similar if switching to 1 kG reference panels. This is based on the observation that the yield of well-imputed high-frequency variants is comparable to our experiences (not shown).

We investigated imputation accuracy by comparing genotypes of masked SNPs with their posteriori distributions after different imputation scenarios. We only masked SNPs of good quality to ensure that error of measured genotypes is as small as possible. Several measures of comparisons of measured and imputed genotypes were considered, namely best-guess genotypes, SEN score and Hellinger score. While Hellinger score measures the agreement of measured and posterior genotype distribution, SEN score is maximal if their expectations coincide [29]. We also studied the software specific quality measure MaCH-Rsq and IMPUTE-info score which however are only defined for entire SNPs rather than single genotypes. An important result of our study is that these measures critically depends on the reference panel used. As a consequence, these scores can predict the imputation accuracy only if the reference panel is genetically similar to the target population. Otherwise even high MaCH-Rsq/IMPUTE-info scores do not guarantee that the estimated genotypes are correct.

To evaluate genetic similarity between different target and reference populations, we computed pairwise *G*_*ST*_, *F*_*ST*_-related scores using our newly developed software fcGENE [27] and SMARTPCA [17] which calculates pairwise *F*_*ST*_^*R*^ between any two populations. The measure *G*_*ST*_ and all of the *F*_*ST*_-related measures were strongly correlated. Relationships are almost linear except for the AfAm population which is a clear outlier. A detailed analysis revealed that *G*_*ST*_ was slightly better correlated with *F*_*ST*_^*WC*^ and *F*_*ST*_^*mWC*^ than with *F*_*ST*_^*R*^. In previous research [20, 45], *G*_*ST*_ and *F*_*ST*_-related scores were estimated for SNPs first and then averaged across all SNPs. However, such type of estimates may not reflect haplotype diversity among populations of different ethnicities [15, 34, 35]. Therefore, we decided to estimate these measures in a haplotype-wise manner averaging their components (i.e. numerator and denominator of the formula) over all SNPs first. Then, the measure is calculated as the ratio of these estimates.

Independent of the type of measures considered, we observed an almost linear relationship between genetic distance and resulting imputation accuracy. Only AfAm showing particularly low imputation accuracy even if using YRI as reference violates this finding. Moreover, even though degree of missingness was shown to be a strong determinant of imputation accuracy [29], the linearity of the above mentioned relationship is preserved for different degrees of missingness. In view of this linear relationship, one can estimate imputation accuracy for a given pair of target and reference population. Relying on Nei’s *G*_*ST*_ we observed satisfactory imputation results for a cut-off of 0.04. Excellent results are achieved if *G*_*ST*_ is less than 0.01. We recommend this threshold for selecting a reference panel at least for medium or small datasets considered in our study. Larger samples of genetically different groups are required for generalization of our result.

Finally, we analysed the performance of genotype imputation for low frequency variants. Although it is known that the imputation of low frequency variants is particularly difficult [46, 47], it has become important in the context of next-generation sequencing. Imputation quality of these variants is much lower than for high-frequency variants. Still we found a negative trend between genetic distance and imputation quality which however is less pronounced than for the high-frequency variants. Interestingly, besides the ethnic similarity, the number of polymorphic sites in the reference panels influences imputation accuracy of low-frequency variants.

As mentioned previously, imputation accuracy is not solely determined by the genetic similarity between the reference and target population. An example is the AfAm population showing lower accuracy than expected on the basis of the genetic distance. The reason is the more complex haplotype structure and generally reduced levels of linkage disequilibrium in African populations which is not measured by the genetic distance [20]. Additional populations of African ancestry are required to analyse this issue and its impact on the relation of genetic similarity and imputation accuracy in more detail.

## Conclusion

We conclude that *G*_*ST*_ and other measures of genetic similarity of homogenous target and reference populations are good predictors of imputation accuracy for imputation frameworks relying on best-matched reference panels. An almost linear relationship of *G*_*ST*_ and various measures of imputation accuracy was observed with exception of the African-American population considered. In our data, excellent imputation results are achieved if *G*_*ST*_ is less than 0.01. However, this threshold might not hold for African populations for which reduced linkage disequilibrium is a stronger determinant of imputation accuracy. For low-frequency variants, the same trend between *G*_*ST*_ and imputation quality was observed, but here, panels with higher number of monomorphic sites (i.e. CHB-JPT) perform below the average. The software specific measures MaCH-Rsq or IMPUTE-info score must be interpreted with caution if the genetic distance of target and reference population is high.

## Additional file

Additional file 1:
**Supplementary Table S1 and Supplementary Figures S1-S11.**

## Abbreviations

GWA: Genome wide association
GWAS: Genome wide association studies
F_ST_: *F*-statistics
G_ST_: *G*-statistics
GQ: Good-quality
HQ: High-quality
MAF: Minor allele frequency
CR: Call rate
EEU: East European populations
SASI: South Asians
EU: Europeans
Jap: Japanese
MEX: Mexican
SEU: South European
AfAm: African-Americans
