# Supplementary material for: Impact of genetic similarity on imputation accuracy
Source: BMC Genet. 2015 Jul 22;16:90. doi: 10.1186/s12863-015-0248-2 (PMC4509609; doi:10.1186/s12863-015-0248-2)
Supplement: Additional file 1: — Supplementary Table S1 and Supplementary Figures S1-S11. [file 12863_2015_248_MOESM1_ESM.docx]

**Impact of Genetic Similarity on Imputation Accuracy**

Nab Raj Roshyara^1,2^ and Markus Scholz^1,2^

1. Institute for Medical Informatics, Statistics and Epidemiology, University of Leipzig, Haertelstrasse 16-18, 04107 Leipzig, Germany
2. LIFE Center (Leipzig Interdisciplinary Research Cluster of Genetic Factors, Phenotypes and Environment), University of Leipzig, Philipp-Rosenthal Strasse 27, 04103 Leipzig, Germany

**MaCH commands:**

**Step1:
./mach1 -p / target_data.ped –d target_data.dat -s hapmap3_ref.snps -h hapmap3_ref.hap.gz --greedy**

**-r 100 --prefix target_data_output_step1**

**Step2:
./mach1 -p target_data.ped -d target_data.dat -s hapmap3_ref.snps -h hapmap3_ref.hap.gz
--crossover target_data_output_step1.rec--errormap target_data_output_step1.rec –greedy**

**--geno --quality --dosage --probs --phase --mle --mldetails --prefix target_data_output_step2**

**MaCH-minimac Commands:**

**Step1:**

**/mach1 -p target_data.ped -d target_data.dat --rounds 100 --states 200 --phase --interim 5 --sample 5 --prefix target_output_step1**

**Step2:**

**./minimac --refSnps hapmap3_ref.snps --refHaps hapmap3_ref.hap --snps snplist_name.txt --haps target_output_step1.hap --round 20 --states 200 --phased --probs --gzip --em --prefix target_output_step2**

**IMPUTE2 Commands:**

**./impute2 -m hapmap3_genetic_map.txt -h hapmap3.hap -l hapmap3.leg -g target_data.gens\**

**-strand_g target_data_strand.txt -pgs -int lowerBound upperBound -Ne 20000 -o target_output_name**

| **Pop 1** | **Pop 2** | **Type of Fst** | **ALL SNPs** | **MAF>=0.05** | **MAF>=0.1** | **MAF>=0.2** |
| --- | --- | --- | --- | --- | --- | --- |
| AfAm | CHB.JPT | Reich FST | 0.097 | 0.151 | 0.154 | 0.153 |
| AfAm | CHB.JPT | Nei Gst | 0.1476 | 0.1461 | 0.1465 | 0.1439 |

**Supplementary Table S1: Impact of low-frequency variants on measures of genetic distance. We present estimates of Reich FST, Nei Gst between Popres subsample AfAm and reference panel CHB.JPT for different cut-offs of minor allele frequency. While Reich FST changes significantly if low-frequency variants are omitted, Nei GST is robust.**

**
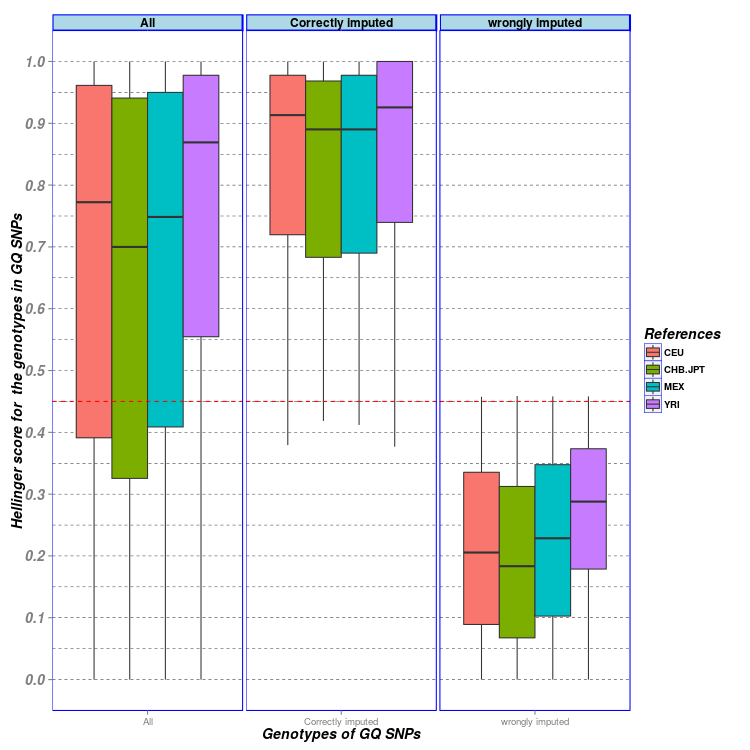
**

**Supplementary Figure S1:** **Box plot of Hellinger scores of target population AfAm obtained from MaCH- imputation with four different reference panels. Results for correctly and wrongly imputed SNPs based on best-guess genotypes are presented separately. CEU achieves highest Hellinger scores for all, correctly and incorrectly imputed genotypes, i.e. performed best among reference panels. As one can see, applying a threshold of 0.45 for Hellinger scores almost ensures that the best-guess genotype is correct.**

**
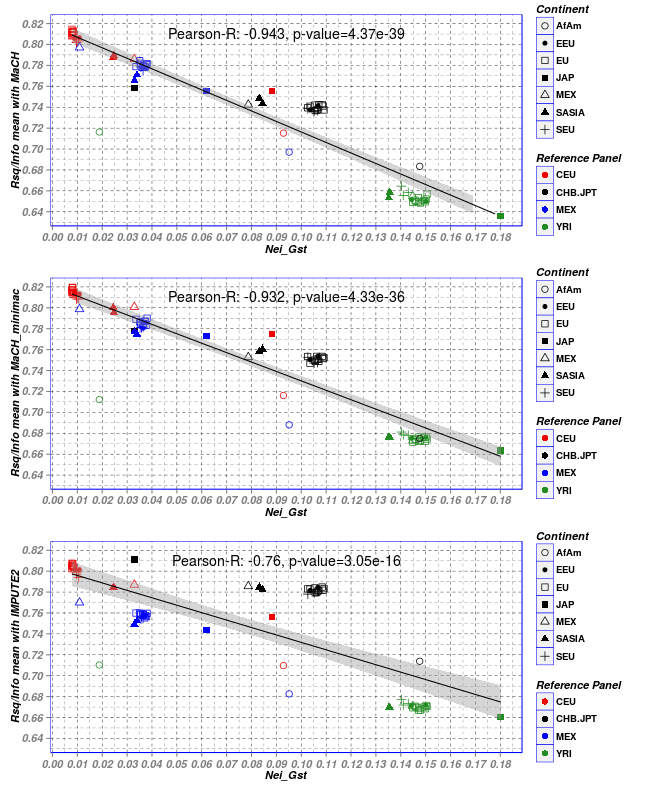
**

**Supplementary Figure S2: Scatter plot of Nei’s** $\mathbf{G}_{\mathbf{ST}}$ **and Average MaCH-Rsq/IMPUTE-info scores of GQ SNPs for the three different software considered. Color decodes reference panel while symbol represents the POPRES population considered. Pearson’s correlation coefficients and the p-values obtained from computing a test of the correlation being zero are also described.**

**
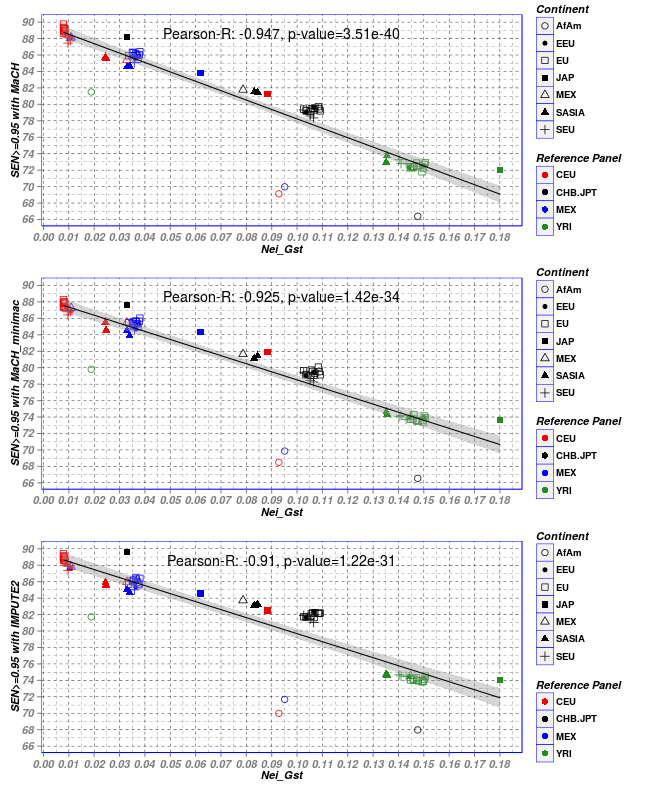
Supplementary Figure S3: Scatter plot of Nei’s** $\mathbf{G}_{\mathbf{ST}}$ **and percentages of gentoypes with good SEN score (SEN ≥0.95) for the three different software considered. Color decodes reference panel while symbol represents the POPRES population considered. Pearson’s correlation coefficients and the p-values obtained from computing a test of the correlation being zero are also described.**

**
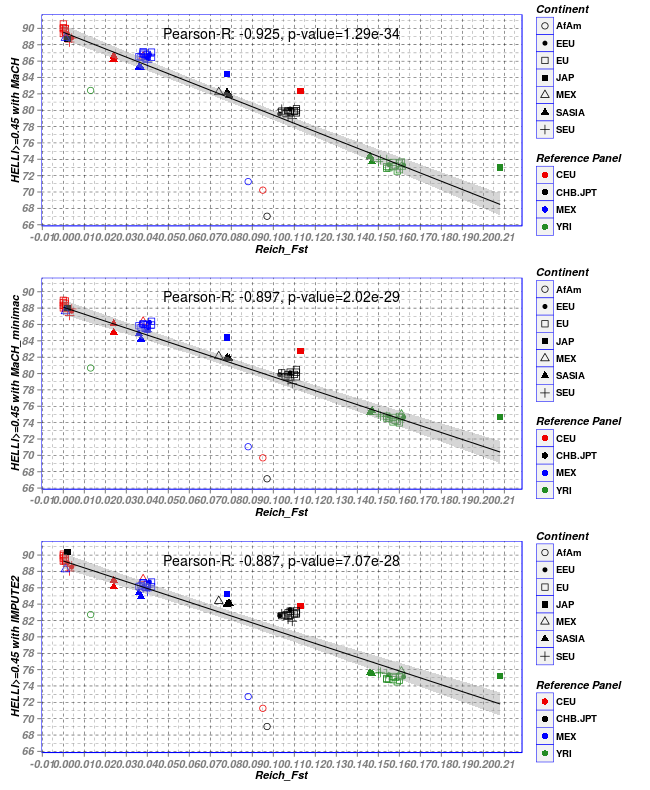
**

**Supplementary Figure S4: Scatter plot of Reich’s** $\mathbf{F}_{\mathbf{ST}}$ **and percentages of gentoypes with good Hellinger score (HELLI ≥0.45) for the three software considered. Color decodes reference panel while symbol represents the POPRES population considered. Pearson’s correlation coefficients and the p-values obtained from computing a test of the correlation being zero are also described.**


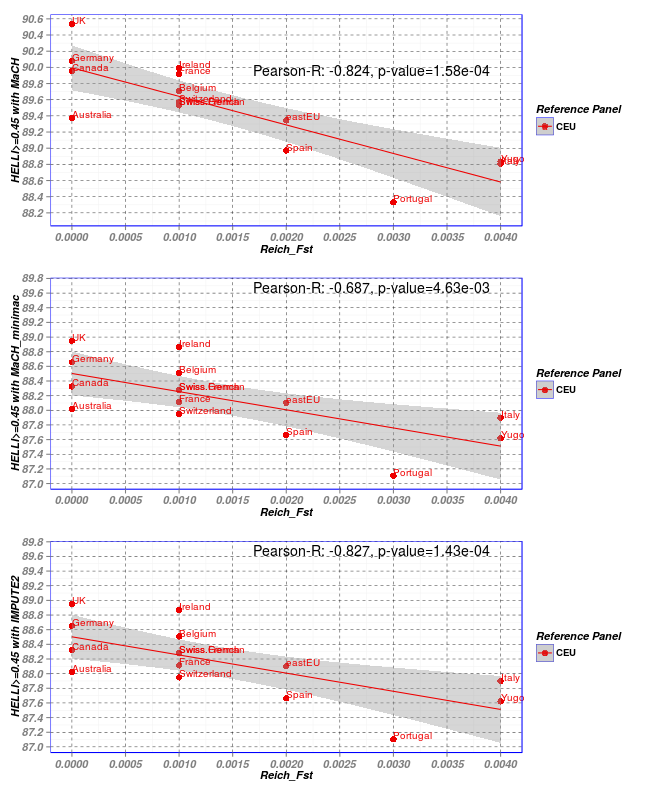


**Supplementary Figure S5: Scatter plot of Reich‘s** $\mathbf{F}_{\mathbf{ST}}$ **and percentages of genotypes with good Hellinger score (HELLI≥0.45 ) for the three software considered. Only European populations are displayed. Color decodes reference panel while symbol represents the POPRES population considered. Pearson’s correlation coefficients and the p-values obtained from computing a test of the correlation being zero are also described.**

**
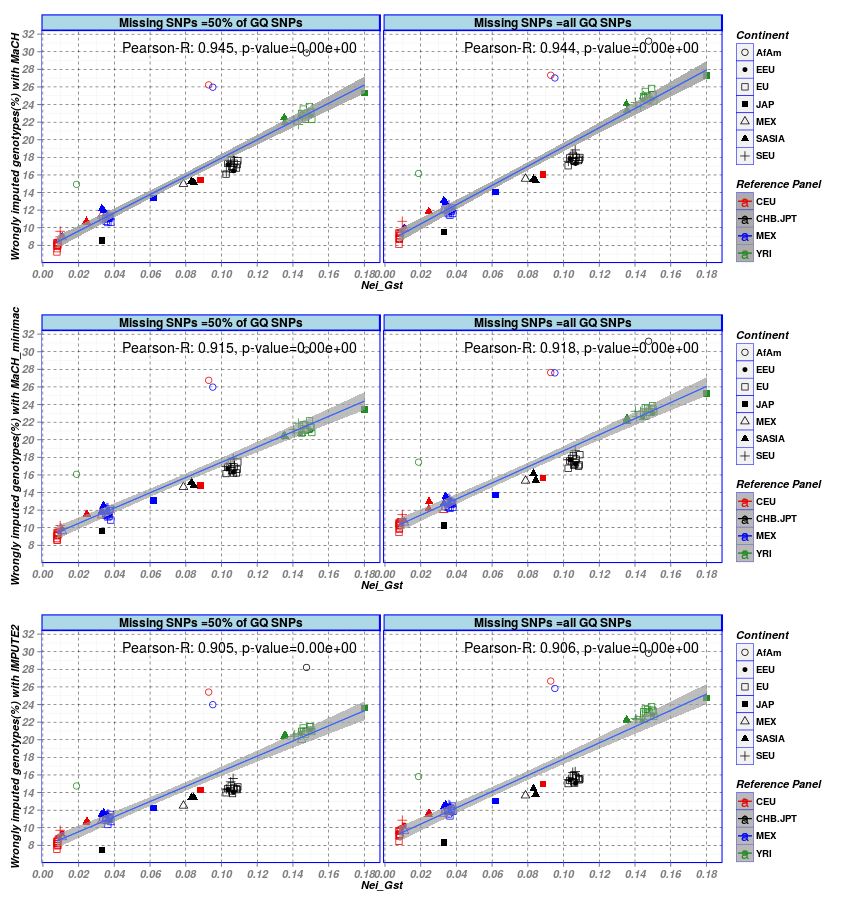
**

**Supplementary Figure S6: Scatter plot of** $\boldsymbol{G}_{\boldsymbol{ST}}$ **and percentages of wrongly imputed genotypes (based on best-guess genotypes) at different degrees of missing. It turns out that degree of missingness has a clear impact on imputation accuracy but the linear trend between** $\boldsymbol{G}_{\boldsymbol{ST}}$ **and imputation accuracy is essentially preserved. Pearson’s correlation coefficients and the p-values obtained from computing a test of the correlation being zero are also described.**


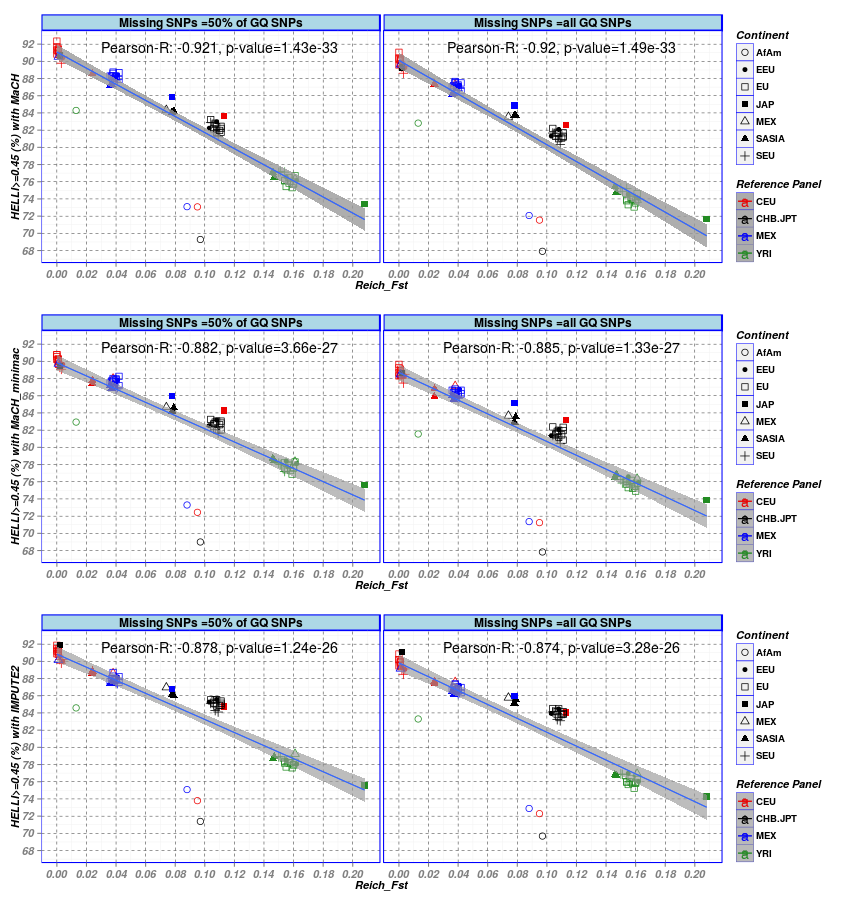


**Supplementary Figure S7: Scatter plot of** $\boldsymbol{F}_{\boldsymbol{ST}}$ **and percentages of genotypes with good Hellinger score (HELLI≥0.45 ). Pearson’s correlation coefficients and the p-values obtained from computing a test of the correlation being zero are also described.**

**
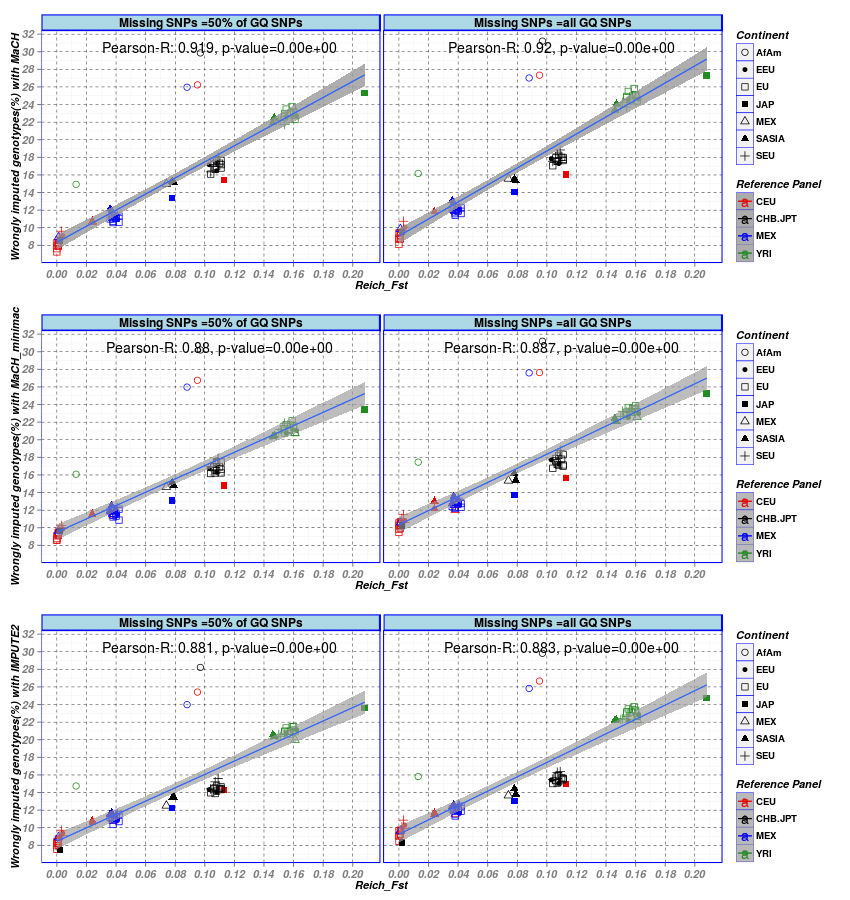
**

**Supplementary Figure S8: Scatter plot of** $\boldsymbol{F}_{\boldsymbol{ST}}$ **and percentages of wrongly imputed genotypes (based on best-guess genotypes) at different degrees of missing. It turns out that degree of missingness has a clear impact on imputation accuracy but the linear trend between** $\boldsymbol{F}_{\boldsymbol{ST}}$ **and imputation accuracy is essentially preserved. Pearson’s correlation coefficients and the p-values obtained from computing a test of the correlation being zero are also described.**

**
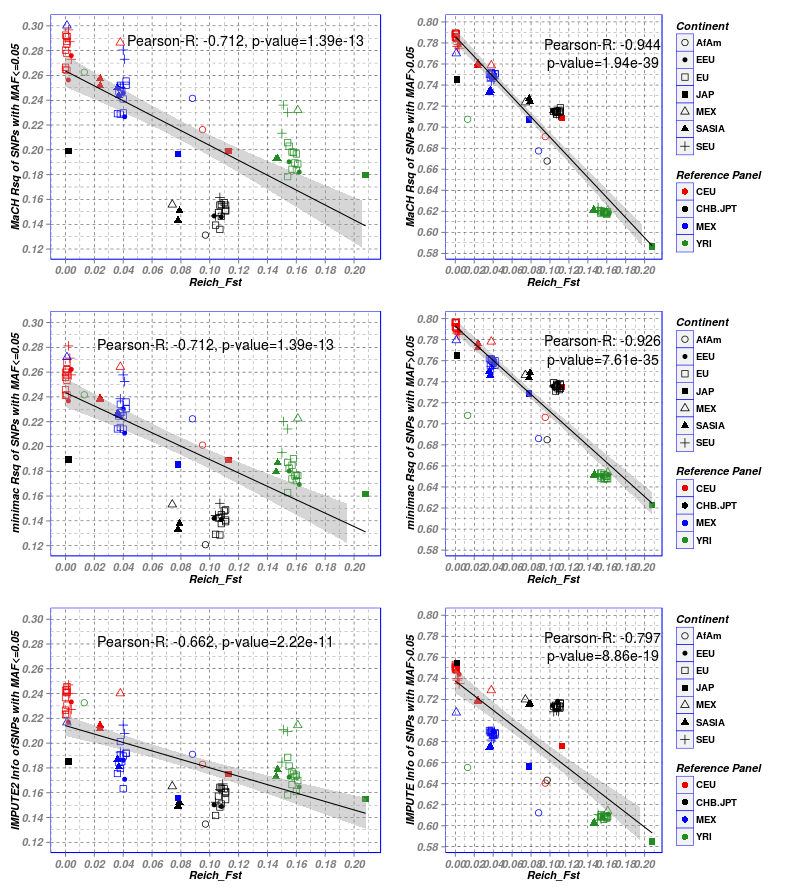
**

**Supplementary Figure S9: Scatter plot of** $\boldsymbol{F}_{\boldsymbol{ST}}$ **and average Rsq-score/Info-score of low-**

**frequency variants (left panels) versus common variants (right panels). We present the results of the three imputation frameworks MaCH, MaCH-minimac and IMPUTE2. For low-frequency variants, both, overall yield of well-imputed SNPs and correlation between** $\boldsymbol{F}_{\boldsymbol{ST}}$ **and imputation accuracy are reduced. Pearson’s correlation coefficients and the p-values obtained from computing a test of the correlation being zero are also described.**

**
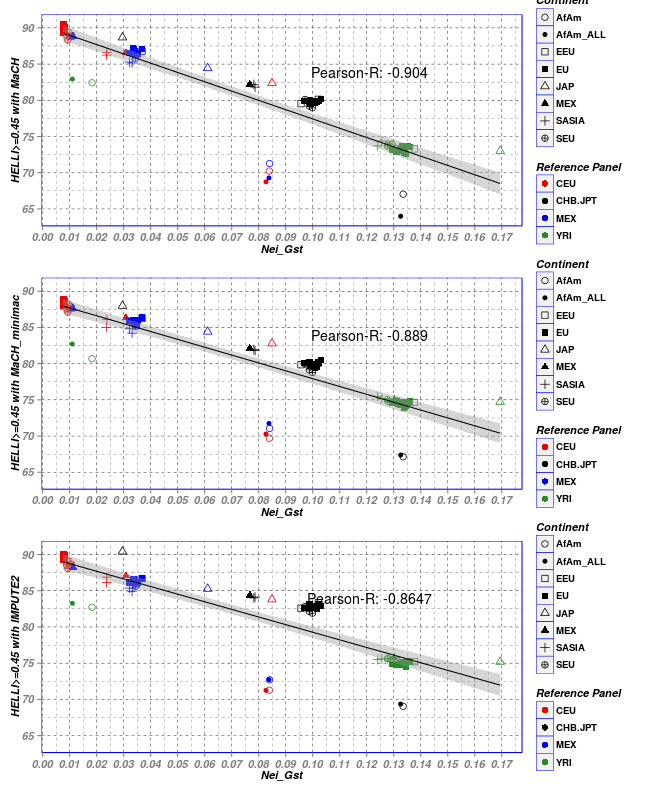
**

**Supplementary Figure S10: Scatter plot of Nei’s** $\boldsymbol{G}_{\mathbf{ST}}$ **and percentages of genotypes with good Hellinger score (HELLI ≥0.45) for the three software considered. Color decodes reference panel while symbol represents the POPRES population considered. In the Figure one extra target dataset AfAm_ALL including all available POPRES samples is also considered.**

**
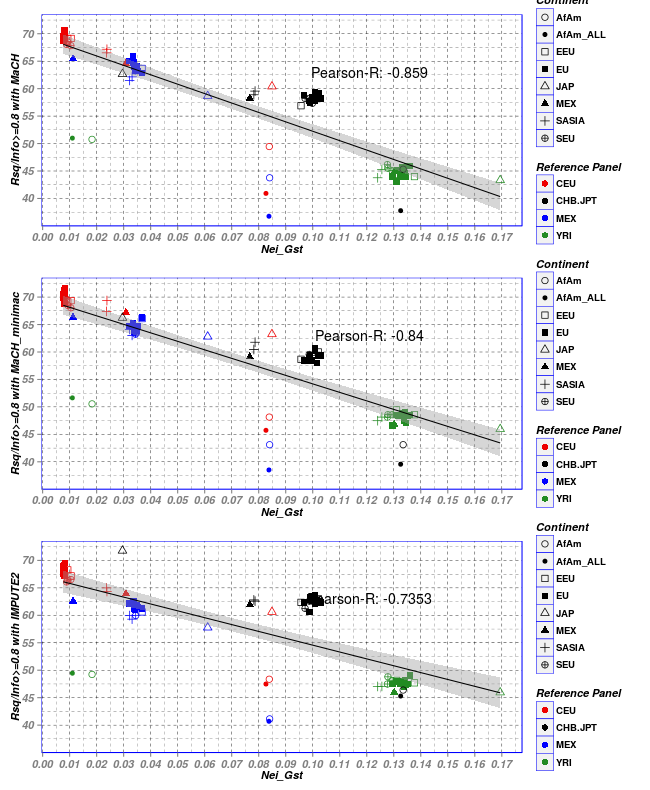
**

**Supplementary Figure S11: Scatter plot of Nei’s** $\boldsymbol{G}_{\mathbf{ST}}$ **and percentages of genotypes with software specific score (MaCH-Rsq≥0.80/ IMPUTE-info≥0.80) for the three software considered. Color decodes reference panel while symbol represents the POPRES population considered. In the Figure one extra target dataset AfAm_ALL including all available POPRES samples is also considered.**
